# The Effect of Illumination and Time of Day on Movements of Bobcats (*Lynx rufus*)

**DOI:** 10.1371/journal.pone.0069213

**Published:** 2013-07-08

**Authors:** Aimee P. Rockhill, Christopher S. DePerno, Roger A. Powell

**Affiliations:** 1 Fisheries, Wildlife, and Conservation Biology, North Carolina State University, Raleigh, North Carolina, United States of America; 2 Department of Biology and Fisheries, Wildlife, and Conservation Biology, North Carolina State University, Raleigh, North Carolina, United States of America; University of Pretoria, South Africa

## Abstract

Understanding behavioral changes of prey and predators based on lunar illumination provides insight into important life history, behavioral ecology, and survival information. The objectives of this research were to determine if bobcat movement rates differed by period of day (dark, moon, crepuscular, day), lunar illumination (<10%, 10 - <50%, 50 - <90%, >90%), and moon phase (new, full). Bobcats had high movement rates during crepuscular and day periods and low movement rates during dark periods with highest nighttime rates at 10-<50% lunar illumination. Bobcats had highest movement rates during daytime when nighttime illumination was low (new moon) and higher movement rates during nighttime when lunar illumination was high (full moon). The behaviors we observed are consistent with prey availability being affected by light level and by limited vision by bobcats during darkness.

## Introduction

Hunting is a delicate balance of energy expended for unit of reward, and many factors affect timing of hunting activities by predators and prey [1].To optimize catch per unit effort, predators concentrate hunting efforts to periods of the day when prey are most active and readily available [2]–[5]. Abiotic factors affecting predator – prey interactions were given limited attention until the 1940s when researchers began investigating the impact of moon phase and lunar illumination on animal movements, habitat use, and predator-prey relationships [6]–[8]. Many mammalian prey studies focused on illumination effects on foraging strategies, and optimality models predicted that prey species would shift habitat use and alter movement rates to minimize predation risk [7], [9]–[13]. Likewise, predators optimize foraging by shifting habitat use, movement rates, and foraging time to maximize hunting success [14], [15].

Generally, small mammals have low predation risk during new moons and high predation risk during full moons [6],[9],[10],[12],[13],[16]–[22]. Therefore, to reduce risk, nocturnal prey behave in ways that reduce vulnerability to predators during high nighttime illumination. During full moon periods, prey reduce movement rates, shift activity periods, reduce food consumption, forage for short periods, spend more time in dense habitat compared to open habitat, and reduce sizes of their foraging areas compared to during new moon periods [7], [9], [10], [12], [13], [16]–[19], [21]–[29]. Although extreme behavioral changes are documented in prey, few studies have investigated the effects of lunar illumination on predator behavior [2], [3], [5].

Changes in foraging behavior by prey, to reduce detection by predators, decreases hunting success for predators [3], [26]. Moon phase can have the second largest effect, after prey species, on hunting success of predators [2], [5]. Further, a study on red foxes (*Vulpes vulpes*) reported individual foxes selected the same prey species during the same moon phases and shifted prey selection when moon phase changed [3]. To counteract changes in activity of prey and to increase hunt success, predators must concentrate hunting efforts during low illumination nights [4]or shift prey selection during high illumination nights towards prey species that may not offer as high of reward (i.e. smaller size) but may be easier to catch.

Although bobcats are commonly thought to be nocturnal or crepuscular, their eyes are proportionately smaller and less well adapted to low light compared to strictly nocturnal cats, allowing bobcats to hunt during day and night [30]–[34]. A majority of bobcat movement studies report peaks in crepuscular activity with highest movement rates at dusk [35]–[38]. Factors affecting bobcat movement rates include temperature, age, sex, and season [35]–[38]. Further, laboratory studies suggest that bobcats locomotion is inhibited by low light and darkness [39]. Thus, hunting may be less successful for bobcats if prey are most active and likely to use open areas during extreme darkness (e.g., new moon periods with no lunar illumination). Previous reports of bobcats' limited night vision, combined with changes in prey availability, suggest a functional response may exist where the use of a prey species by a bobcat depends on the prey species availability and catchability. Throughout their range, bobcats' diets include a variety of prey that are active throughout different times of the day [1], [31], [40]–[42]. Specific to coastal North Carolina, bobcats' diet includes prey that are active diurnally (birds, eastern gray squirrels, *Sciurus carolinensis*), late afternoon to midnight (cotton rats, *Sigmodon hispidis*), night, dawn, and dusk (rabbits, *Sylvilagus* spp., northern raccoons, *Procyon lotor*) and throughout the 24 hour day (white-tailed deer, *Odocoileus virginianus*) [40].

Bobcats are solitary hunters adapted for short bouts of speed and either stalk and ambush or actively search their home ranges for prey [31]. Active home range searches often occur when prey densities are low and result in high movement and activity rates by bobcats [43]. Given depressed activity of small prey during high lunar illumination, the abilities of bobcats to hunt throughout the day, and reports that bobcat are most active when prey are active, we hypothesized that bobcats shift movement rates with changes in illumination. Therefore, we tested 3 hypotheses: 1) movement rates of bobcats increase with increases in lunar illumination but decrease with an increased availability of sunlight; 2) movement rates of bobcats increase with increasing percentages of lunar illumination (<10, 10 - <50, 50 - <90, and >90); and 3) bobcats have low movement rates during new moon nights and high movement rates during full moon nights.

## Materials and Methods

**Figure 1:**
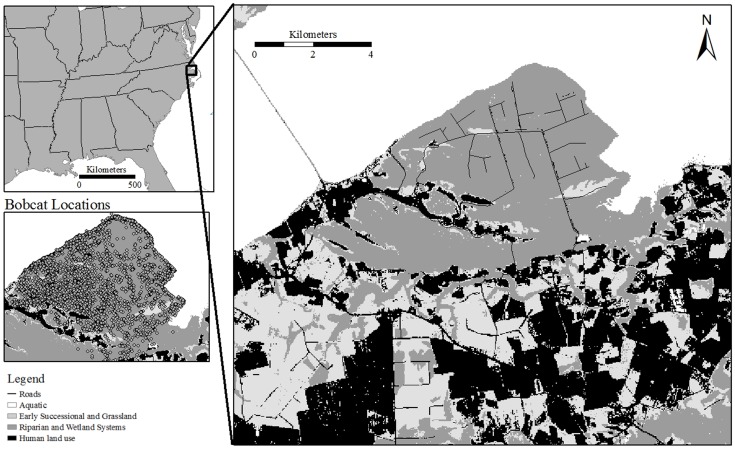
Bull Neck Swamp Research Forest, North Carolina, 2009. The white dashed line represents the property boundary; however, bobcat GPS locations were acquired on the surrounding property.

We studied bobcats at Bull Neck Swamp Research Forest (henceforth ‘Bull Neck’, Figure 1), a 25-km^2^ wetland located on the southern side of Albemarle Sound in Eastern North Carolina (35° 57′ S, 76° 25′ E). The property was one of the largest remaining tracts of undeveloped waterfront on North Carolina's Albemarle Sound, containing more than 11 km of undisturbed shoreline and 10 km^2^ of preserve. Bull Neck had 5 land cover types: non-riverine swamp forest, peatland Atlantic white cedar (*Chamaecyparis thyoides*), mesic mixed hardwood forest, tidal cypress gum swamp, and tidal freshwater marsh. Initial results from 132 vegetation surveys showed high variation between survey locations with the same land cover classification (e.g., visual obstruction values that ranged from 17 to 100 percent in the non-riverine swamp forest land cover class, unpublished data). Due to high within land cover class variation of vertical structure, we used the broadest land cover class available from the Southeast Gap Analysis Project (SE-GAP, Level 1) to assess the potential for analyzing habitat use variation in relation to illumination [44]. We assumed prey availability was similar to that reported by King *et al*. who examined stomach contents of 389 trapper harvested bobcats in coastal North Carolina [40]. Percent occurrence of the most selected food items included: rabbits, 52.8%; birds, 33.2%; cotton rats, 15%; white-tailed deer, 13.4%, eastern gray squirrels, 8.1%; northern raccoons, 5.2%, and voles (*Microtus* spp.), 3.3% [40]. Further, through extensive mammal surveys (scent station, camera, trapping, spotlight) and 3 years of visual observations on the property, we confirmed the presence of marsh rabbits (*S. palustris*), eastern cottontails (*S. floridanus*), cotton rats, white-tailed deer, eastern gray squirrels, northern raccoons, Virginia opossums (*Didelphis virginianus*), feral pigs (*Sus scrofa*), American beavers (*Castor canadensis*), nutrias (*Myocastor coypus*), voles (*Microtus spp.*), and numerous waterfowl and land birds on the property (A. P. Rockhill, unpublished data). Mean monthly temperatures ranged from 6.5 C° in January to 26.6 C° in July and rainfall averaged 126.5 cm per year [45]. The property was managed by the Fisheries, Wildlife, and Conservation Biology Program at North Carolina State University, Raleigh, North Carolina.

During 1–11 March 2008 and 8–22 March 2009, we live-trapped bobcats using #1.5 Victor, padded-jaw, foot-hold traps. We immobilized bobcats with an intramuscular injection of Ketamine (10 mg/kg) and Xylazine (0.75 mg/kg) or Ketamine (4 mg/kg), Medetomidine (40 mcg/kg), and Butorphanol (0.4 mg/kg) [46]. We fitted each bobcat with a GPS collar weighing 250 g (Televilt, Lindesberg, Sweden). Also, the collars broadcast in the VHF range so that bobcats could be located from the ground. Immobilized bobcats were reversed with Yohimbine (0.2 mg/kg) or Atipamezole (0.2 mg/kg), depending on the anesthetizing protocol. All animal handling techniques were approved by the Institutional Animal Care and Use Committee at North Carolina State University (08-012-O) and followed guidelines provided by the American Society of Mammalogists [47] and ASAB/ABS Guidelines for the Use of Animals in Research. All efforts were made to minimize suffering.

Each GPS collar collected a location every 2 h beginning at 18:00 and ending at 06:00 with an additional location taken at 12:00 each day. Also, on the 1^st^ and 15^th^ of each month, the collars collected a location every hour from 1:00–24:00. To ensure that collars functioned properly and that study animals were still alive; we monitored bobcats weekly using VHF telemetry equipment. We used Hawth's Tools 3.27 [48] in ArcGIS 9.2 to calculate linear distances moved by bobcats between consecutive locations and divided distances by times between locations to estimate movement rates (n = 5,924). Movement rates can be used to estimate general patterns of daily activity accurately with results similar to analysis using net activity time and percent locations with activity [49].

Initial regression tests with independent variables produced significant effects of temperature and standardized time and we blocked by these variables to meet assumptions of independence. Temperature and time were grouped to the nearest degree and hour and treated as discrete variables. Further, sex or individual bobcats did not have a significant effect but we treated the latter variable as a random effect to correct for the lack of independence. We blocked by these variables when testing our 3 hypotheses. We used analysis of covariance (ANCOVA) with logarithmically transformed movement rates (hereafter “rate”, to meet assumptions of normality) as our dependent variable. We used Tukey's Studentized Range (HSD) mean comparison tests with a Tukey-Kramer adjustment for multiple comparisons to compare mean rates for all analyses. All analyses were conducted using SAS (Cary, North Carolina) and alpha level was set at P = 0.05.

To quantify illumination levels throughout each 24-hour day, we acquired daily sunrise, sunset, moonrise and moonset times from the Naval Oceanography Portal (http://www.usno.navy.mil/). We partitioned each 24-hour day into day (1 h post sunrise – 1 h pre sunset), crepuscular (2 h periods surrounding sunrise and sunset), and nighttime (1 h post sunset – 1 h pre sunrise) periods. We further grouped nighttime locations into moon (hours between moonrise and moonset) and dark (new moon or nighttime hours before moonrise and after moonset) categories. Rates between day, crepuscular, moon, and dark were compared using ANCOVA (n = 5,924).

We assigned each nighttime location a lunar illumination value based on the fraction of the moon that was illuminated and on moonrise and moonset times (http://www.usno.navy.mil/). Lunar illumination values ranged from 0.1 to 1 and hours of the night with no moon received a moon illumination value of zero. For example, if moonrise was at 08:20, moonset at 23:08, and 16% of the moon was illuminated then hours 09:00–23:00 were assigned an illumination value of 0.16. We then grouped illumination levels into 4 categories that included <10%, 10 - <50%, 50 - <90%, and >90% [23]. We tested the subset of data consisting of nighttime only locations (n = 3,073) using ANCOVA. Previous research has noted variation in lunar illumination based on moon angle and cloud cover and suggests illumination could be increased with high, thin clouds and bias would be added to analysis by making assumptions of decreased illumination based on cloud cover alone [50], [51]. Further, consistencies were lacking between local weather at Bull Neck and weather conditions recorded at the 2 closest weather stations. Often, Bull Neck had rain when surrounding areas had clear skies. Therefore, we did not include cloud cover to avoid adding bias to assigned illumination values.

To test for temporal shifts in movement rates, we compared hourly rates between new and full moon periods. Using a subset of the nighttime only data (n = 2,246), we assigned each hourly location as either new (n = 1,103) or full (n = 1,143) moon for 5 days surrounding each period. For example, if a new moon occurred on 08 March, then all hourly rates from 01:00 on 06 March – 24:00 on 11 March were assigned the new moon period. Because locations were acquired throughout an 8 month period, we transformed times of each day so that sunrise occurred at 06:00 and sunset at 18:00. Transformations were made with the following equations:

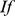
 0< time ≤ rise *then* newtime  =  time * 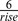

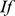
 rise < time ≤12 then newtime  = 6+ (time – rise) * 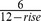

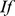
 12< time ≤ set *then* newtime  = 12+ (time – 12) * 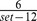

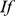
 time > set *then* newtime  = 18+ (time – set) * 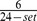

where; time  =  the actual time a GPS location was taken, rise and set  =  daily sunrise and sunset times acquired from the Naval Oceanography Portal (http://www.usno.navy.mil/), and newtime  =  the transformed standardized time. The transformed standardized time variable allowed us to analyze hourly movement rates based on illumination instead of time of day.

## Results

We trapped 9 individual bobcats in 2008 (4 females, 3 males) and 2009 (3 females, 1 male) during 725 and 1,291 trap nights, respectively. Of the 9 bobcats, 2 adult females were recaptured in 2009. We collected 6,647 GPS locations from 5 (2008; 3 females, 2 males) and 2 (2009; 2 females) bobcats between March and October of 2008 and 2009. Of the 4 bobcats that remained on the property, 99% of the GPS locations were in the Riparian and Wetland Systems land cover class (Figure 1). Data from 2 dispersing juveniles in 2008 indicated high use of Human Land Use areas that were likely crop fields pine plantations in various stages of growth that provided cover for dispersal. Insufficient bobcat locations in various land cover classes that covered multiple levels of illumination prevented us from assessing potential changes in habitat use based on time of day or illumination.

Movement rates differed by period (daytime, crepuscular, moon, no moon; n = 5,924, F_3_ = 2.78, P = 0.03) with highest movement rates during crepuscular (153 m/hr) and day (144 m/hr) periods and lowest movement rates during the dark period (no moon; 120 m/hr, Table 1). On average, bobcats moved more during crepuscular periods than during dark periods (P<0.001, Table 1).

**Table 1:** Mean movement rates (m/hr), standard deviation (SD), and Tukey's Studentized Range (HSD) mean comparison groupings for bobcats at Bull Neck Swamp Research Forest, North Carolina from 2008–2009.

		Movement Rate (m/hr)	SE	Tukey's HSD Grouping
Period	Dark	120	6	A
	Moon	140	5	A,B
	Crepuscular	153	5	B
	Day	144	7	B
Lunar	<10%	119	6	B
	10 - <50%	161	11	A
	50 - <90%	145	8	A,B
	>90%	122	8	B

Movement rates are shown for Period analysis and Lunar Illumination analysis.

Movement rates during night differed by lunar illumination period (n = 3,073; F_3_ = 5.26, P = 0.001). Bobcats had higher movement rates (+42 m/hr) when illumination was 10 - <50% than when illumination was <10% (P<0.0001, Table 1). Illumination levels <10% and 50 - <90% (P = 0.8617) and 50 - <90% and >90% (P = 0.7848) were similar and no difference was detected between low (<10%) and high (>90%) illumination (P = 1.0000, Table 1).

**Figure 2:**
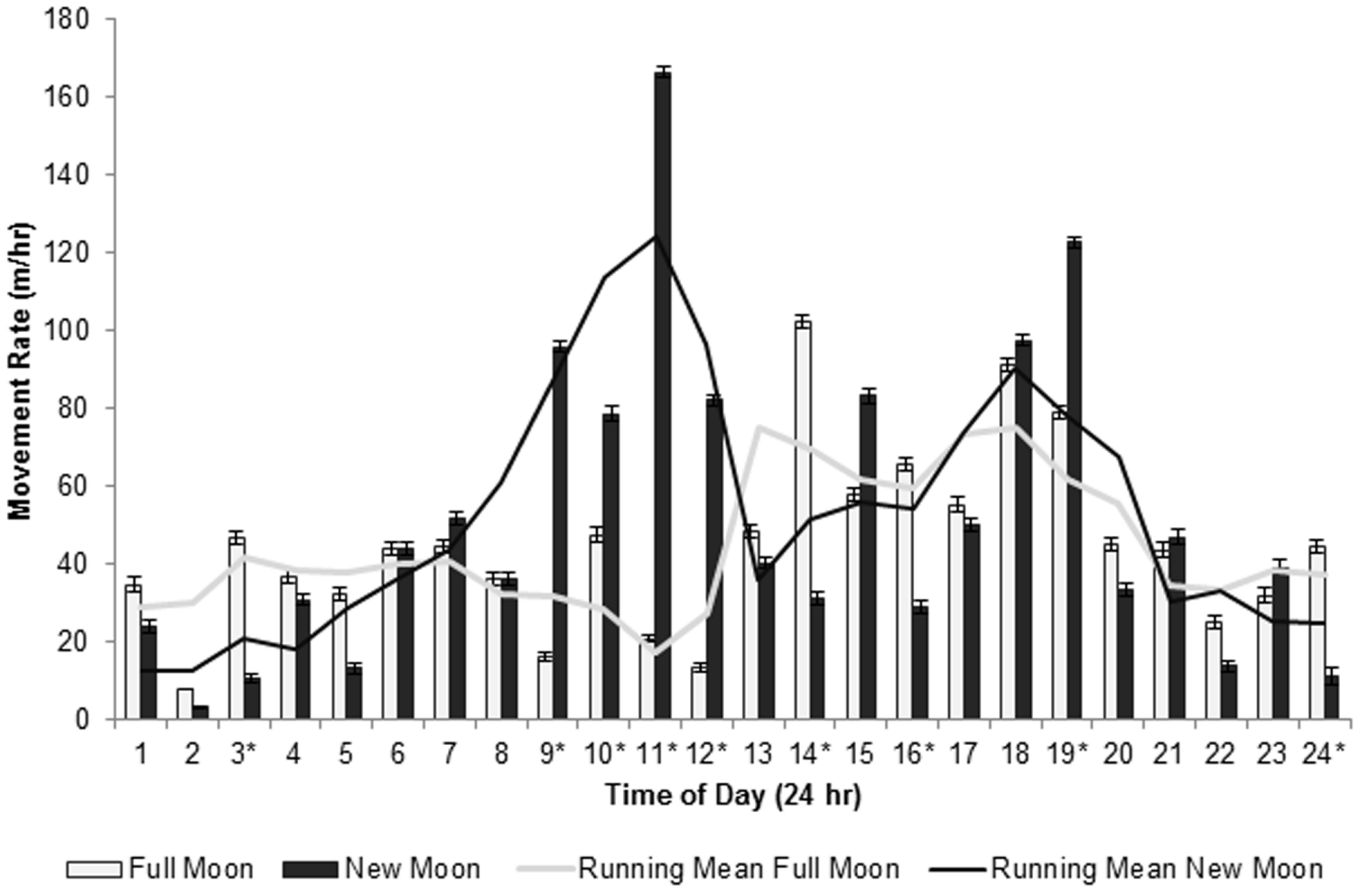
Mean hourly movement rates (± standard error) by bobcats during full and new moon periods. Movement rates were averaged for 5 bobcats at Bull Neck Swamp Research Forest, North Carolina from March 2008 and October 2009 and include 5 days surrounding new moon or full moon periods for each month. Lines indicate the running average mean for movement rates during new (gray line) and full (black line) moon periods. An * above the Time of Day indicates a significant difference (P<0.05) in movement rates between new and full moon periods.

Standardized time affected bobcat movement rates (n = 2,246, F_1_ = 38.93, P<0.001) and bobcats had high movement rates from 9:00–12:00 (late morning) and again from 14:00–19:00 (mid-afternoon through dusk; Figure 2). On average, bobcats moved 17 m/hr more during full moon nights than during new moon nights (n = 2,246, F_1_ = 3.884, P = 0.028). During new moon periods, bobcats exhibited low movement rates (<40 m/hr) during nighttime hours and high movement rates (>80 m/hr) during most daytime hours (Figure 2). Conversely, during full moon periods bobcats had low movement rates (<40 m/hr) during the early daytime hours with increases to 166 m/hr in the afternoon hours (Figure 2).

**Figure 3:**
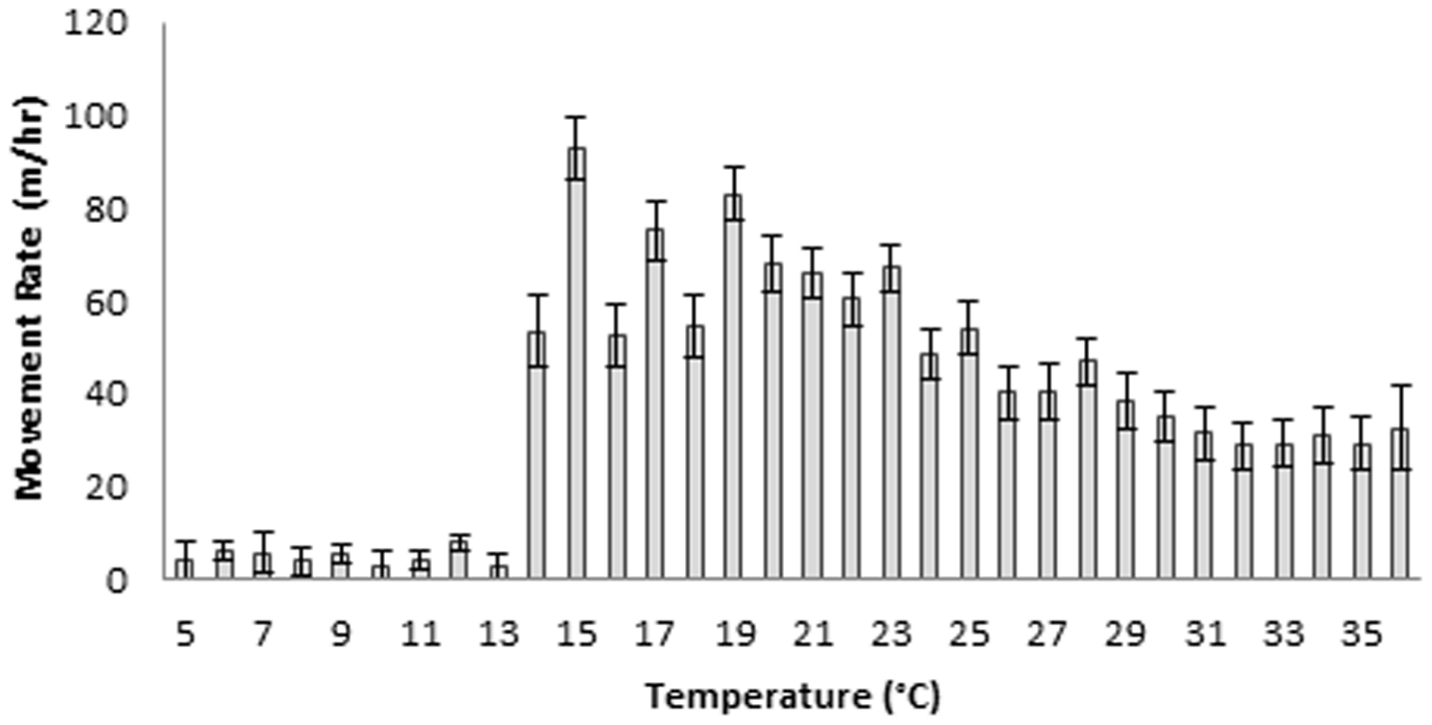
Mean movement rates (m/hr) per 1° change in temperature by bobcats at Bull Neck Swamp Research Forest, North Carolina, March 2008–October 2009. Temperature was averaged using the temperature at start and end points used to calculate mean movement rates. Capped lines represent standard error bars.

Bobcat was not a significant variable (df = 1, F = 0.39, P = 0.53). Temperature (n = 5,924, df = 1, F = 148.68, P = <0.0001) was the best predictor of bobcat movement rates; bobcats moved <10 m/hr below 14°C and >50 m/hr between 15–25°C (Figure 3) and interacted with period of the day (df = 3, F = 9.63, P = <0.0001).

## Discussion

Bobcats can be flexible in their circadian rhythms and can adjust foraging time to track their prey, as can other mammalian predators who can fast longer than several hours [14], [15]. During our study, bobcats adjusted their movement rates with changes in illumination associated with moon phase and time of day. Bobcats had 44% higher movement rates during crepuscular periods compared to moon periods and our results support the hypothesis that if prey move and forage less during high lunar illumination [6], [7], [28], then bobcats must search larger areas to meet energy requirements during such periods. The high movement rates of bobcats during high illumination implies bobcats are not able to take advantage of increased prey movement during dark periods and may hunt prey that are available during crepuscular or daylight hours to compensate for poor night vision. Analysis based on period indicated that bobcats moved more during moonlit periods than during dark periods. Further analysis revealed the higher movement rate during moon periods was driven by periods of lunar illumination of 10 to 49%. We hypothesize that 10 to 49% lunar illumination represents an optimal nocturnal hunting time when small prey have high movement rates [7] yet illumination is enough to facilitate efficient hunting. Although we were unable to use cloud cover to infer changes in illumination, we do recognize that our results may be masked by assuming higher illumination than available if clouds were present. We hypothesize that direct on-the-ground measurements of illumination would produce more statistically significant results between illumination categories.

Movement peaks at dusk are similar to those previously reported [35]–[38]. We hypothesize that bobcats have high movement rates during early evening because prey are available and diverse, and because illumination levels are still high enough for bobcats to see well [34]. It is important to understand the physiological limitations of predators in different systems and, perhaps more importantly, how predators compensate for limitations. Clearly, bobcat vision is well suited for diurnal foraging [30], [32], [33] and a high daytime movement rate during dark nights suggests compensation for poor night vision when no lunar illumination is available. Analyzing the movement rates by illumination and lunar cycles allowed us to identify diel shifts where movement peaks occurred during mid-day. Zezulak and Schwab [38] reported diel shifts in bobcat activity from crepuscular winter movements to nocturnal spring movements, and hypothesized the shift was due to high temperature (>26°C) or reduced prey activity. Our data supports their hypothesis of a diel shift due to high temperature and shows a similar decrease in activity around 25°C. Accounting for the effect of temperature in our analysis suggests that illumination and prey activity drive bobcat movement rates which highlight the importance of incorporating temperature and seasonal variation in future lunar phase and illumination analysis of bobcat movement.

Our research highlights the risk of losing critical data by averaging movements over periods of time (i.e., days, months, seasons). For example, bobcats moved 17 m/hr more during full moon periods than during new moon periods. While a difference of 17 m/hr seems minimal, it is important to remember this movement rate was averaged over 5 days surrounding the full and new moon. When the data were separated into hourly intervals, we were able to identify hours with up to 140 m/hr differences that reflected the significant results. While past results may mislead researchers to schedule GPS collars to collect data only during crepuscular hours, we hope this study emphasizes the importance for 24 hour data collection. Further, we would like to caution the reader of the possibility of lower movement rates with increased amounts of time between locations (e.g., 1 hour versus 6 hours) that may occur if individuals turn back on themselves. While we understand that GPS technology can preclude data collection at shorter intervals, we recommend acquiring fixes at frequent intervals. This is especially important with species that forage frequently (e.g., white-tailed deer). Nevertheless, it is essential that researchers incorporate temporal variables in analysis. Averaging movement rates over a daily or seasonal period will cause researchers to miss important insights to predator hunting strategies.

We hypothesize that predators shift habitat use based on lunar illumination to compensate for shifts reported in habitat use by prey [4], [7], [9], [12], [17], [19], [22], [25]. Our results support the hypothesis that prey species forage less in open areas during high lunar illumination to decrease the risk of predation [7], [12], [18]. Further, if prey species are more likely to be detected by predators during high lunar illumination [6], [7], [28], predators are more vulnerable to visual detection by their prey. We hypothesize that an increased risk of visual detection combined with decreased prey use of open areas during high lunar illumination would cause a shift in habitat use to interior forests. Unfortunately, the homogeneity of land cover across our study area prevented us from investigating shifts in habitat use. However, our research indicates that illumination and the population dynamics of prey should be built into habitat models, leading to 4 dimensional (or more) habitat maps (the 4^th^ dimension being lunar phase or illumination).

That bobcat match activity within the combination of solar and lunar cycles to availability of prey may make bobcats appear cathemeral. Their circadian behaviors, however, are hardly random. Predation is a multispecies dynamic incorporating predators and prey; the hunting strategy of a predator undoubtedly guides the prey response to risk, while prey foraging behavior undoubtedly guides the hunting strategy of the predator. Ultimately, our study highlights the importance of incorporating illumination into movement and habitat use analysis of all animals, ideally where diverse habitats exist. Funston reporting moon phase as the second most important variable in lion (*Panthera leo*) hunt success, second to prey species [1]. Although her study focused on a predator, as did ours, it is equally important to simultaneously record illumination and time of day effects on prey (i.e., impala (*Aepyceros melampus*), buffalo (*Syncerus caffer*), kudu (*Tragus strepsiceros*)) so we can begin to understand the behavioral plasticity of predators and their prey and make informed decisions on landscape scale management. Including movement dynamics of predators and prey will provide the insights needed to understand why and when predators use habitats [52]–[55].
